# The Accuracy of MRI in the Local Staging of Endometrial Cancer: An Experience From a Tertiary Care Oncology Institute in Pakistan

**DOI:** 10.7759/cureus.31053

**Published:** 2022-11-03

**Authors:** Palwasha Gul, Khanda Gul, Muhammad Omer Altaf, Ainy Javaid, Javeria Ashraf

**Affiliations:** 1 Department of Radiology, Bolan Medical Complex Hospital, Quetta, PAK; 2 Department of Radiology, Shaukat Khanum Memorial Cancer Hospital and Research Centre, Lahore, PAK; 3 Department of Obstetrics and Gynaecology, Bolan Medical Complex Hospital, Quetta, PAK

**Keywords:** magnetic resonance imaging, invasion, accuracy, histopathology, endometrial cancer

## Abstract

Objective

Our objective is to assess the diagnostic accuracy of contrast-enhanced magnetic resonance imaging (MRI) in identifying the depth of myometrial invasion and cervical stromal involvement in endometrial carcinoma (EC) along with nodal status and its correlation with surgical and histopathological (HP) findings.

Materials and methods

We performed a retrospective study on female patients at Shaukat Khanum Memorial Cancer Hospital and Research Centre, Lahore, Pakistan. Patients with endometrial carcinoma (CA) were searched from the electronic record system, and a total of 188 patients fulfilling the study criteria were selected. All the patients were evaluated using a 1.5T MRI and underwent a hysterectomy. The outcome of preoperative MRI was correlated with histopathology results, keeping pathology as the gold standard.

Results

A total of 188 patients were included in the study, with a mean age of 56.67 ± 12.47 years. Of the patients, 72 (38.3%) were diagnosed with stage 1a. The second common stage was 1b, seen in 43 (22.9%) patients. It was found that the staging of endometrial CA on MRI and HP were significantly correlated for myometrial invasion (stage 1a and 1b), cervical stromal involvement (stage 2b), serosal and adnexal (stage 3a), vaginal (stage 3b), and nodal (stage 3c) involvement as shown by their p-values of <0.01. However, in cases of parametrial invasion (stage 3b), bladder involvement, and rectal involvement (stage 4), MRI showed decreased sensitivity as shown by their p-values of 0.833, 0.87, and 0.9, respectively.

Conclusion

Preoperative MRI can predict local disease and low-risk patients accurately, thereby helping in proper surgical planning and avoiding more extensive surgery such as lymphadenectomy in these patients.

## Introduction

Endometrial carcinoma (EC) is the most prevalent gynecological malignancy in the Western world [1]. It makes up 6% of all cancers in females internationally; however, in developing countries, it has an incidence of 5.9 per 100,000 females. EC is diagnosed mainly during the sixth and seventh decades of life [2,3]. As the majority of females present early due to postmenopausal bleeding, the 10-year survival rate is 75%. Multiple risk factors are linked to EC, such as obesity, high body mass index (BMI), unopposed estrogen intake, nulliparity, polycystic ovarian syndrome, tamoxifen therapy (selective estrogen receptor modulator), diabetes mellitus, and hereditary nonpolyposis colorectal cancer.

Two types of EC are described based on histopathology (HP) and clinical outcome, type I being the commonest, the majority (80%-85%) being endometrioid adenocarcinoma. It is estrogen driven and has a good prognosis. Type II constitutes 10%-15% EC, predominantly serous carcinomas (CA), which are more aggressive with a poor prognosis.

Imaging modalities including sonography and computed tomography (CT) can be used for the evaluation of EC; however, magnetic resonance imaging (MRI) is a superior modality, giving a more detailed overview, and hence helps better in treatment planning and prognosis [1,3].

Females complaining of postmenopausal bleeding are initially evaluated with transvaginal ultrasound (TVUS), which is used to measure endometrial thickness. TVUS has a few pitfalls; it is highly dependent on the operator, it cannot demarcate the tumor margins if it is diffusely infiltrating into the myometrium [3,4], and it has a limited field of view [5]. CT also shows less sensitivity and specificity in correctly determining local invasion as opposed to MRI [3].

The International Federation of Gynecology and Obstetrics (FIGO) system is used for EC staging. It was revised in 2009, simplifying stage I and II endometrial cancer, reducing the substages from three to two in stage I, and removing cervical mucosal invasion as a separate substage. Depending on the histopathological tumor type, grade, and myometrial invasion, EC patients are divided into high- and low-risk categories [1].

## Materials and methods

Study design, setting, and duration

We performed a retrospective study on female patients at Shaukat Khanum Memorial Cancer Hospital and Research Centre, Lahore, Pakistan. Data from the last three years (January 2017 to December 2019) were searched, and a total of 188 patients fulfilling the study criteria were selected. All the patients were evaluated using a 1.5T MRI and underwent a hysterectomy.

Inclusion criteria

Patients were diagnosed with either carcinoma of the endometrium after endometrial sampling or ultrasonography (USG) suspicion of carcinoma of the endometrium.

Exclusion criteria

Patients with metastatic disease who were not candidates for surgery were excluded.

Data collection procedure

The hospital’s ethical review committee granted an exemption for this retrospective study (exemption number EX-11-12-19-01). Patients with endometrial CA were searched from the electronic record system. A comparison was made between MRI results and histopathology outcomes for early and advanced endometrial carcinoma, keeping histopathology results as the gold standard. Statistical analysis was performed using Statistical Package for the Social Sciences (SPSS) (IBM SPSS Statistics, Armonk, NY, USA).

MRI technique

Imaging was performed in the supine position on a partially filled bladder. To reduce peristalsis, patients fasted for five hours before imaging with 20 mg intramuscular (IM) hyoscine administration. Axial, axial oblique, and sagittal fast spin-echo T2-weighted images (T2WI) and axial T1-weighted images (T1WI) of the pelvis were obtained. Dynamic post-contrast imaging was performed with an injection of 0.2 mmol/kg gadolinium-diethylenetriamine pentaacetate (DTPA) at a rate of 2 cc/kg. Diffusion-weighted MRI of the pelvis was performed with different b-values.

## Results

**Figure 1:**
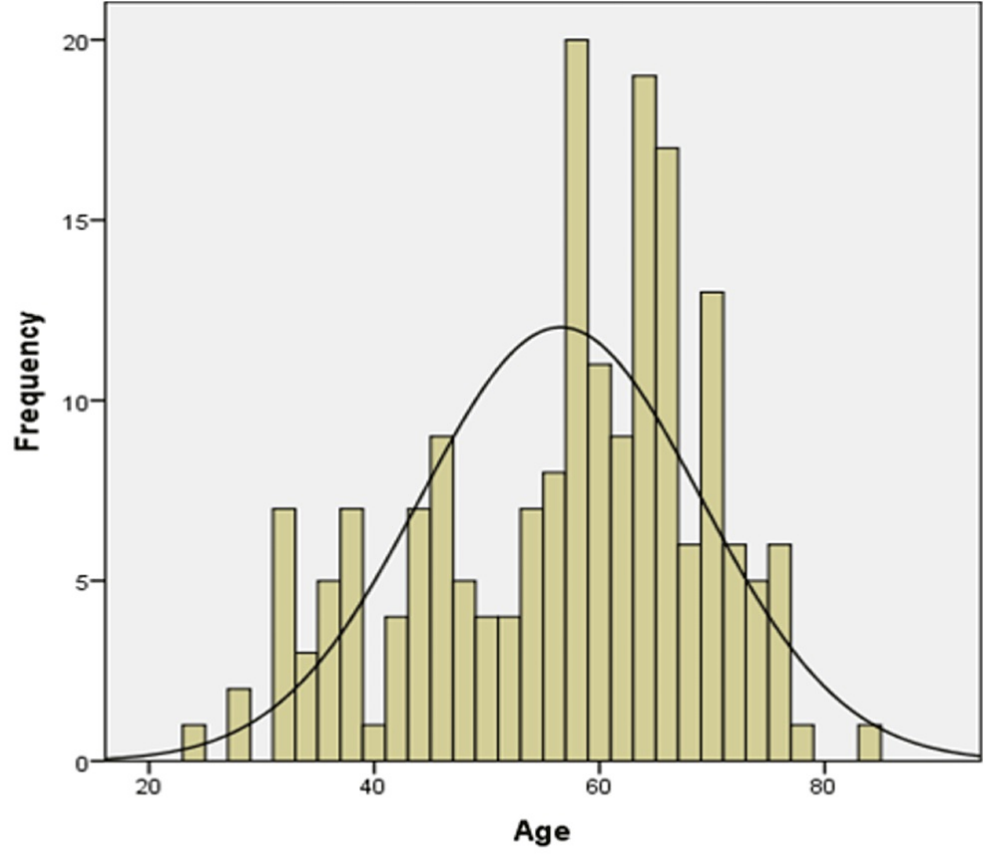
Histogram showing the frequency of endometrial carcinoma against age with a mean age of 56.56 years and a standard deviation of 12.47 years.

Our study included a total of 188 patients diagnosed with EC. The average age at presentation is 56.56 years with a standard deviation (SD) of 12.47 years as shown by the histogram in Figure 1.

Around 72/188 (38.3%) patients were diagnosed with stage 1a. The second common stage was 1b, seen in 43 (22.9%) patients. The relative frequencies of other stages are shown in Table 1.

**Table 1:** Relative frequencies of stages as shown on MRI and HP. MRI: magnetic resonance imaging, HP: histopathology

		Staging on histopathology													
		Stage 1a		Stage 1b		Stage 2		Stage 3a		Stage 3b		Stage 3c		Stage 4a	
		Number	%	Number	%	Number	%	Number	%	Number	%	Number	%	Number	%
Staging on MRI	Stage 1a	72	38.3%	8	4.3%	3	1.6%	0	0%	0	0%	0	0%	1	0.5%
	Stage 1b	20	10.6%	43	22.9%	2	1.1%	1	0.5%	3	1.6%	1	0.5%	0	0%
	Stage 2	5	2.7%	2	1.1%	10	5.3%	0	0%	0	0%	0	0%	0	0%
	Stage 3a	0	0%	4	2.1%	1	0.5%	2	1.1%	0	0%	0	0%	0	0%
	Stage 3b	3	1.6%	1	0.5%	0	0%	0	0%	1	0.5%	0	0%	0	0%
	Stage 3c	1	0.5%	1	0.5%	1	0.5%	0	0%	0	0%	1	0.5%	0	0%

The number of patients diagnosed with stage 1a and stage 1b on MRI and correspondingly on histopathology are shown in Table 2. A total of 156 (83%) patients showed similar staging on both MRI and histopathology, showing a significant correlation (p-value < 0.01).

**Table 2:** Number and percentage of patients with less than and more than half of myometrial involvement on MRI and correspondingly on HP (p-value < 0.01). MRI: magnetic resonance imaging, HP: histopathology

Myometrial invasion		Pathology			
		Equal to or more than half of myometrium invasion (stage1b)		Less than half of myometrial invasion (stage 1a)	
		Number	%	Number	%
MRI	Equal to or more than half of myometrium invasion (stage1b)	74	39.4%	23	12.2%
	Less than half of myometrial invasion (stage 1a)	9	4.8%	82	43.6%

**Figure 2:**
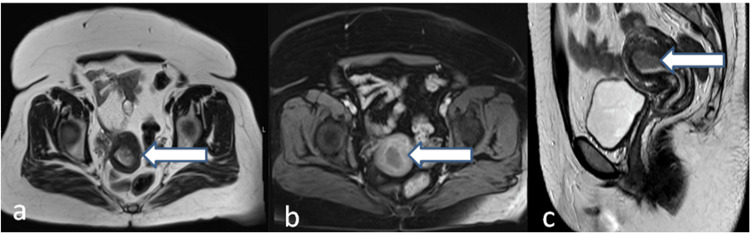
MRI of the pelvis showing thickened endometrium measuring 17 mm with intermediate signals on axial and sagittal T2WI (a and c). Minimal myometrial invasion (<50%) is seen on sagittal T2WI (c) with disruption of the junctional zone. Minimally enhancing endometrial tissue (b). No pelvic lymphadenopathy. MRI was staged as T1a N0. MRI: magnetic resonance imaging, T2WI: T2-weighted imaging

Figure 2 shows stage Ia disease on MRI.

**Figure 3:**
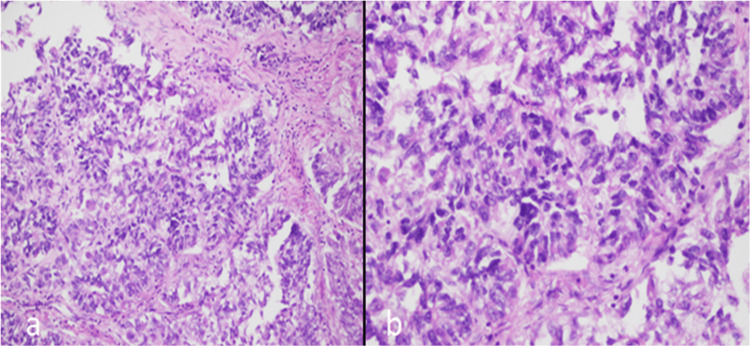
High-power images (20× (a) and 40× (b)) showing moderate atypia and atypical mitotic figures in endometrial carcinoma.

Figure 3 shows high-power images with moderate atypia and atypical mitotic figures in endometrial carcinoma.

**Figure 4:**
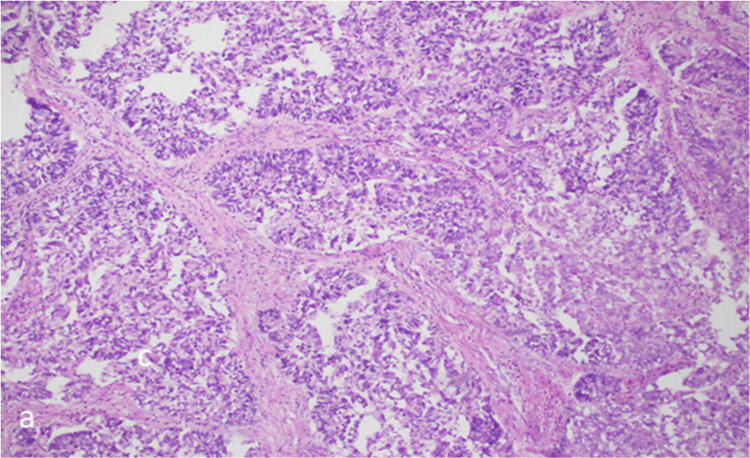
Lower-power image (a) showing back-to-back glands lacking intervening stroma in endometrial carcinoma.

Figure 4 shows a lower-power image with back-to-back glands lacking intervening stroma in endometrial carcinoma. 

Cervical stromal involvement (stage II) was absent in 156 (83%) patients on both MRI and histopathology. MRI and histopathology showed a significant correlation (p-value < 0.01) for detecting cervical stromal involvement (Table 3).

**Table 3:** Number and percentage of patients with cervical stromal involvement (stage II) on MRI and correspondingly on HP (p-value < 0.01). MRI: magnetic resonance imaging, HP: histopathology

Cervical stromal involvement		Pathology			
		Absent		Present	
		Number	%	Number	%
MRI	Absent	156	83%	9	4.8%
	Present	12	6.4%	11	5.9%

Serosal and adnexal involvement (stage IIIa) were absent in 184 (97.9%) and 181 (96.3%) patients, respectively, on both MRI and histopathology, showing a significant correspondence in both diagnostic entities (p-value < 0.01), as shown in Table 4 and Table 5.

**Table 4:** Number and percentage of patients with serosal involvement (stage IIIa) on MRI and correspondingly on HP (p-value < 0.01). MRI: magnetic resonance imaging, HP: histopathology

Serosal invasion		Pathology			
		Absent		Present	
		Number	%	Number	%
MRI	Absent	184	97.9%	1	0.5%
	Present	2	1.1%	1	0.5%

**Table 5:** Number and percentage of patients with adnexal involvement (stage IIIa) on MRI and correspondingly on HP (p-value < 0.01). MRI: magnetic resonance imaging, HP: histopathology

Adnexal involvement		Pathology			
		Absent		Present	
		Number	%	Number	%
MRI	Absent	181	96.3%	5	2.7%
	Present	1	0.5%	1	0.5%

Similarly, Table 6 and Table 7 demonstrate the absence of vaginal and parametrial involvement with high true positive rates of 96.8% (182) and 95.2% (179) of patients, respectively. It was seen that eight patients were suspected of parametrial involvement on MRI, but when followed on histopathology, none of these showed significant parametrial involvement. Thus, the low sensitivity of MRI to detect parametrial involvement is shown by an insignificant p-value (p-value = 0.833).

**Table 6:** Number and percentage of patients with vaginal involvement (stage IIIb) on MRI and correspondingly on HP (p-value < 0.01). MRI: magnetic resonance imaging, HP: histopathology

Vaginal involvement		Pathology			
		Absent		Present	
		Number	%	Number	%
MRI	Absent	182	96.8%	3	1.6%
	Present	2	1.1%	1	0.5%

**Table 7:** Number and percentage of patients with parametrial involvement (stage IIIb) on MRI and correspondingly on HP (p-value = 0.833). MRI: magnetic resonance imaging, HP: histopathology

Parametrial involvement		Pathology			
		Absent		Present	
		Number	%	Number	%
MRI	Absent	179	95.2%	1	0.5%
	Present	8	4.3%	0	0%

Table 8 shows nodal involvement (stage IIIc) on MRI and histopathology. Of the patients, 95.7% showed no nodal involvement on both MRI and histopathology, whereas three (1.6%) patients showed nodal involvement on MRI, which was seen in the pathology report later. Similarly, four (2.1%) patients showed false positivity of nodal involvement on MRI.

**Table 8:** Number and percentage of patients with nodal involvement (stage IIIc) on MRI and correspondingly on HP (p-value = 0.005). MRI: magnetic resonance imaging, HP: histopathology

Nodal status		Pathology			
		No		Positive pelvic nodes (stage 3c1)	
		Number	%	Number	%
MRI	No	180	95.7%	3	1.6%
	Positive pelvic nodes (stage 3c1)	4	2.1%	1	0.5%

Rectal and bladder involvement are shown in Table 9 and Table 10, respectively, showing a significant correspondence in MRI and histopathology. None of the patients had rectal and bladder involvement on MRI; however, it was seen in one patient on histopathology.

**Table 9:** Number and percentage of patients with rectal involvement (stage IV a) on MRI and correspondingly on HP (p-value = 0.87). MRI: magnetic resonance imaging, HP: histopathology

Rectal involvement		Pathology			
		Absent		Present	
		Number	%	Number	%
MRI	Absent	187	99.5%	1	0.5%

**Table 10:** Number and percentage of patients with bladder involvement (stage IVa) on MRI and correspondingly on HP (p-value = 0.9). MRI: magnetic resonance imaging, HP: histopathology

Bladder involvement		Pathology			
		Absent		Present	
		Number	%	Number	%
MRI	Absent	187	99.5%	1	0.5%

## Discussion

MRI has superior soft tissue resolution and can precisely decide the pretreatment local staging of endometrial cancer. The staging accuracy of MRI is reported to be 83%-92% [2]. EC shows hypo- to isointense signals on T1-weighted imaging (T1WI) and intermediate signals compared to normal endometrium on T2-weighted imaging (T2WI). It enhances less than the myometrium on post-gadolinium dynamic imaging [2,3,6].

MRI is the best imaging modality for assessing the extent of myometrial and cervical involvement. This in turn correlates with tumor grade, lymph node (LN) involvement, and prognosis. On MRI, intact low-signal intensity junctional zone and a smooth uninterrupted band of early sub-endometrial enhancement on T2 and dynamic contrast-enhanced (DCE) images would rule out myometrial involvement. A depth of <50% or ≥50% of the myometrial involvement is classified as a stage Ia/Ib tumor [1,7-9].

T2WI is used to assess cervical stromal invasion, which is suggested by the replacement of low cervical stroma signals by intermediate tumoral signals. However, care should be taken to assess if there is only a tumor bulge into the cervical canal or invasion of the stroma. Diffusion restriction on diffusion-weighted imaging (DWI) and apparent diffusion coefficient (ADC) and interrupted normal enhancement of the cervical stroma on dynamic contrast-enhanced (DCE) imaging suggest cervical stromal invasion [1,7,8].

The involvement of the ovary and vagina suggests stage IIIa and IIIb tumors. DWI and DCE imaging are specifically useful in diagnosing the involvement of the cervix and vagina by small implants [1,10]. Rectal and bladder wall invasion are well assessed in sagittal images. Advanced stage such as IVa is excluded by the presence of a fat plane between the tumor and adjacent structures [1,11].

Assessing lymph node (LN) involvement has a crucial role in tumor staging, treatment planning, and prognosis. Stage IIIc1 represents pelvic node involvement, and stage IIIc2 represents para-aortic nodal involvement. Deeper myometrial involvement, higher histopathological grade, and type II tumors are risk factors for nodal involvement. The frequency of nodal involvement increases from 3% to 46% with superficial versus deep myometrial invasion. Tumor location can affect the distribution of lymph nodes. The uterine body and cervix drain into pelvic nodes, while retroperitoneal and common iliac territory nodes drain the upper part of the uterus. Inguinal nodes are non-regional and represent stage IV disease. Nodal size, if more than 1 cm on the short axis, can help differentiate benign and malignant nodes on imaging. The morphological appearance of the lymph node can also help in the assessment of its involvement, such as rounded shape and jagged margins, abnormal signals on T2-weighted imaging, merged appearance, and necrosis in the center. Necrosis in the center of the node is definitive for nodal involvement with a positive predictive value of 100% [1,8,12-15].

The precision of MRI is 55%-77% in diagnosing myometrial invasion. In the evaluation of the presence of myometrial invasion, the accuracy of T2-weighted imaging and dynamic contrast imaging was 67.9% and 84.9%, respectively [16,17]. In a study conducted by Zamani et al., the accuracy of MRI was 90.74% for deep myometrial invasion [18]. Another study has also shown the accuracy of MRI for the depth of myometrial invasion ranging from 83% to 91% [19]. Our study also showed a good correlation between MRI and HP results. The diagnostic accuracy of MRI in diagnosing cervical stromal infiltration was 89.28% [2] and 90%-92% [20].

MRI can help in assessing the stage of EC and deciding the management of these patients. Surgery is the management of choice for early endometrial cancer. Based on staging, cervical stromal involvement, and the depth of myometrial invasion, the type of surgery can differ from a simple hysterectomy without lymphadenectomy to radical surgery with systematic lymphadenectomy. In low-risk patients, avoidance of unnecessary lymphadenectomy is important. The stratification of high- and low-risk patients can be done on the basis of the depth of myometrial invasion, grade, and the histological subtype of the tumor before surgery [18].

## Conclusions

MRI is the imaging modality of choice for the pretreatment evaluation of patients with endometrial carcinoma and has become routine imaging in most centers. Preoperative MRI can predict local disease and low-risk patients accurately, thereby helping in choosing appropriate treatment such as surgical planning and avoiding more extensive surgery such as lymphadenectomy in these patients and hence reducing treatment-related morbidity.
